# Different sound characteristics produced by the left and right pectoral fins constitute a new form of lateralization in a vocal fish

**DOI:** 10.1002/jez.2660

**Published:** 2022-10-10

**Authors:** Isabelle P. Maiditsch, Friedrich Ladich

**Affiliations:** ^1^ Department of Behavioral and Cognitive Biology University of Vienna Vienna Austria; ^2^ Paul Scherrer Institute (PSI) Villigen Switzerland

**Keywords:** agonistic sounds, lateralization, peak‐to‐peak amplitudes, sound‐generating mechanism, temporal sound characteristics, vocal fish

## Abstract

Songbirds and toothed whales are able to produce different sounds with the left and right part of their sonic organs, a phenomenon termed lateralized sound production. In fishes this phenomenon is poorly known, with lateralization having been observed solely in the channel catfish (*Ictalurus punctatus*). They produce more sounds with their right pectoral fins. Croaking gouramis *Trichopsis vittata* beat their pectoral fins alternately, resulting in a series of two‐pulsed sound bursts termed croaking sounds. This study investigates lateralized sound production by comparing temporal and amplitude characteristics of sound bursts generated by pectoral fins in *T. vittata*. Croaking sounds, produced during dyadic contests, were analyzed in 19 females. We investigated the following characteristics of sound bursts: burst period, pulse period within bursts, the relative peak‐to‐peak amplitudes of bursts, and the ratio of peak‐to‐peak amplitudes of the first and second pulse within bursts. Sound bursts produced by the right and left sonic organ differed in 17 out of 19 females in at least one to four measured sound characteristics. The number of females whose temporal characteristics differed between pectoral fins was significantly higher than the number of females lacking such differences (16 out of 19). This was not the case for amplitude characteristics. Our data demonstrated that the sound characteristics produced by the left and right sonic organ in *T. vittata* differed significantly in most specimens. These differences in sound properties may constitute a new form of lateralized sound production in vocal fishes.

## Research Highlights

- Sounds produced by the left and right pectoral fin in croaking gouramis differintemporal sound characteristics.
- Differences between sounds particularly involved temporal sound properties.
- Croaking sounds generated by left and right sonic organs may constitute a new form of lateralized sound production in animals.

## Graphical abstract

Sounds produced by the left and right so nic organ differ in croaking gouramis (*Trichopsis vittata*, Labyrinth fishes).

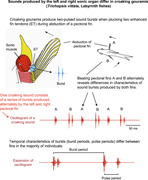

## 1 INTRODUCTION

A preference of one side of the body for a function or an activity is a phenomenon termed lateralization. Lateralization in sound production is the ability of an organism with two sonic organs to use one such organ, i.e. located on the left or on the right side of the body, to produce sounds or merely a particular type of sound more often on the other. Differences in sound production between the left and right side of the body or of vocal/sonic organs have been described in a few nonrelated animal taxa. Although well known in oscine songbirds, this issue is poorly studied in aquatic animals, having been described in toothed whales and in a single fish species.

Songbirds are known to produce a wide variety of sounds ranging from monosyllabic calls to very complex and long songs. The syrinx, the main sound‐producing organ in songbirds, is a bipartite organ in which each bronchus contains an independent sound source at its cranial end (Catchpole & Slater, 1995; Suthers, 2010). Songbirds can sing with either side of their syrinx alone, switch from one side to the other, or sing with both sides simultaneously (Suthers, 1990, 2004). In most birds studied, high frequencies are generated on the right side, low frequencies on the left side, and midrange frequencies can originate on either side (Suthers, 2010). Within marine mammals, which possess a completely different sound‐generating mechanism, a functional difference in both sides of the sonic organs has been described in toothed whales. Echolocation and social sounds are generated in the nasal system through two phonic lip pairs located on the underside of vestibular air sacs below the blowhole (Cranford et al., 1996). A growing body of evidence indicates that the two lip pairs do not work concomitantly in producing a sound but that the right phonic lips are responsible for the production of echolocation clicks and the left pair is used for communication signals (Ames et al., 2020; Madsen et al., 2013).

Lateralization of sound production has apparently been described in only one fish species so far (Fine et al., 1996) and suggested, but not verified, in another one (Fine, 1982). Fishes possess a large variety of sound‐generating mechanisms (Fine & Parmentier, 2015; Ladich & Bass, 2011; Ladich & Fine, 2006). These mechanisms may be subdivided into those involving unpaired sound sources (swimbladder) and those using paired ones (pectoral fins). The swim bladder is vibrated rapidly by fast contracting muscles called drumming muscles. Vibrating the swimbladder involves only one sound source, obviating any lateralization. Sonic motor neurons innervating the left and right swimbladder drumming muscles may discharge synchronously such as in toadfishes (Batrachoidiformes) or asynchronously as described in searobins (Triglidae) by Bass and Baker (1991) and Connaughton (2004). Fine (1982) described a possible lateralization due of drumming sounds in the oyster toadfish *Opsanus tau*. Different contraction patterns of the left and right swimbladder muscles suggested the capability to produce two different outputs, which could be interpreted as a type of lateralization. Subsequent work by the authors (Thorson & Fine, 2002) demonstrated that these unusual sounds were in fact caused by a nearby fish grunting over the calls of its neighbor, termed acoustic tagging, and not by lateralization.

The second group of sonic organs in fishes comprises pectoral fins that enable fins to produce sounds independently from each other. Representatives of numerous catfish families possess an enhanced first pectoral fin ray often named pectoral spine; its dorsal proximal process can be rubbed in a groove of the shoulder girdle, resulting in the emission of broadband stridulation sounds (Fine & Ladich, 2003; Ladich & Maiditsch, 2020; Mohajer et al., 2015; Parmentier et al., 2010). Fine et al. (1996) showed that channel catfish exhibited a fin preference. Nine out of 10 channel catfish preferred the right pectoral fin over the left to produce stridulation sounds when hand‐held in air.

The pectoral sound‐generating mechanism in croaking gouramis differs widely from non‐related catfishes. Croaking gouramis generate pulsed sounds by two enhanced pectoral fin tendons located at the left and right body side (pectoral fins). These tendons are stretched during abduction of fins and plucked over enhanced bony elevations of two fin rays, emitting two pulses per fin (Kratochvil, 1978, 1985; Ladich & Fine, 1992, 2006; Ladich et al., 1992; Liesch & Ladich, 2020). The left and the right pectoral fin are always beaten alternately, yielding a series of double‐pulsed sound bursts known as a croaking sound. The pectoral tendon plucking mechanism is found only in the genus *Trichopsis* within the family Osphronemidae (Kratochvil, 1985), which comprises three species (Ladich et al., 1992; Richter, 1988). In contrast to channel catfish, pectoral fins in croaking gouramis cannot be moved independently from each other during sound production, resulting in a similar number of sound bursts when vocalizing during aggressive interactions between two fish (dyadic contests).

Accordingly, this study was designed to investigate differences in temporal and amplitude characteristics of sound bursts produced by pectoral fins in female *T. vittata*. Females were chosen because of availability and because differences between sexes are small (Ladich & Maiditsch, 2018; Ladich, 2007). Although it was not possible to determine if the left or right pectoral fin generated a particular sound burst within a croaking sound (because opponents were head to tail circling during agonistic interactions), differences in temporal and amplitude characteristics of bursts and pulses within sound bursts could be analyzed.

## 2 METHODS

### 2.1 Animals

Nineteen female *T. vittata* were used for the study (body weight: 0.80–1.56 g, standard length: 35–42.8 mm). Fish were kept in community tanks (100 × 55 × 40 cm) at 25 ± 1°C in a 12 h light–12 h dark cycle. Tank bottoms were covered with sand, flowerpots, and plants as hiding places. Fish were primarily fed food flakes (Tetramin) five times a week. Sexing of fish was based on the presence of the whitish ovary in females (see supplementary figure in Maiditsch & Ladich, 2022). After experiments, fish were returned to the community tanks.

### 2.2 Behavior and sound recordings

**Figure 1:**
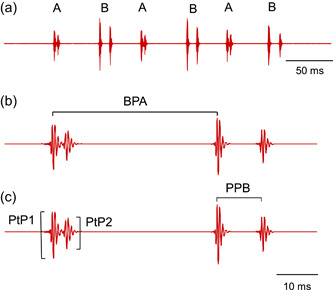
Oscillogram of a female croaking sound consisting of (a) a series of six double‐pulsed bursts produced by pectoral fins A and B alternately and (b and c) enlargement of the last two bursts. (b) Shows burst period of fin A (BPA), and (c) a pulse period of fin B (PPB). Note that the pulse periods (PP) and relative peak‐to‐peak amplitudes (PtP) were larger in fin B than in fin A. PtP1, PtP2 ‐ relative peak‐to‐peak amplitude of the first and second pulse within a double‐pulsed burst of fin A.

**Figure 2:**
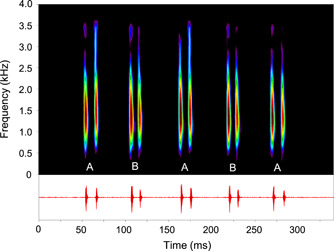
Sonagram and oscillogram of a croaking sound consisting of five double‐pulsed bursts generated by a female *Trichopsis vittata*. Note shorter pulse periods in sound bursts produced by fin B in contrast to fin A. The main energy of the sound is located above 1 kHz. Sampling frequency 44.1 kHz, filter bandwidth 200 Hz, overlap 75%, Hamming window.

Females were isolated for 5 days in one half of isolation tanks (50 × 27 × 30 cm) under conditions similar to the community tanks, to reduce pre‐experience dominance of fish, which may be gained in the community tank. On the fifth day, two females were introduced into the left and right halves of the test tanks (50 × 27 × 30 cm), which were separated by a nontransparent sheet. The test tank was placed on a table that rested on a vibration‐isolated concrete plate. The entire set‐up was enclosed in a walk‐in semi‐sound‐proof room, which was constructed as a Faraday's cage. Dyadic contests started after the separating sheet was removed. The agonistic interaction consisted of erecting unpaired fins, head to tail circling and sound production. Such lateral display bouts (or sequences) were interrupted by air‐breathing (see fig. 2 in Maiditsch & Ladich, 2022). Typically, both fish emitted croaking sounds alternately. The sound‐producing fish could be determined by the rapid pectoral fin beating during which the whole animal was shaking. Nonetheless, it was not feasible to determine if the left or right pectoral fin generated the burst within a croaking sound; bursts could be discriminated by their shape during the analyses and therefore pectoral fin sides are referred to as side A and B (Figures 1 and 2).

Acoustic signals and behavior were recorded using a hydrophone (Brüel & Kjaer 8101, sensitivity: −186 dB re 1 V/μPa) connected to a microphone power supply (Brüel & Kjaer 2804) which was connected to the XLR mic input of a 4‐K video camera (Panasonic HC‐X1000). The entire setup was positioned behind a curtain so that animals could not see the experimenter.

### 2.3 Sound analysis

The video camera recorded LPCM‐coded sounds were afterwards rendered in Sony Vegas Pro 13.0 to WAV‐format (44.1 kHz, 16 bit). To avoid low‐frequency noise and high‐frequency reverberations of aquarium walls, the WAV‐files were high pass filtered (>0.2 kHz) and low pass filtered (<3.5 kHz) in CoolEdit 2000 (Syntrillium Software Corporation) (Akamatsu et al., 2002). Sounds were subsequently analyzed in S_TOOLS‐STX 3.7.8 (Acoustics Research Institute, Austrian Academy of Sciences). Each croaking sound consists of a series of double‐pulsed bursts when pectoral fins are beaten alternately. Each burst is produced by one pectoral fin.

The following sound characteristics were determined (Figure 1):

1. Burst period (BP): The time between the first peaks of the first pulse of two consecutive bursts.

2. Pulse period (PP): The time between the first peaks of two successive pulses within a double‐pulsed burst. No PPs could be determined in single‐pulsed bursts.

3. Peak to peak amplitude of bursts (PtP): The relative amplitude between the highest and the lowest peak of the larger pulse within a burst. The larger pulse is always the first pulse in a double‐pulsed burst.

4. Ratio of the peak‐to‐peak amplitudes of the first and the second pulse (PtP1 and PtP2) within a burst. This ratio within a burst was calculated as PtP1/PtP2.

Differences between sound characteristics and body sides are phrased as burst types.

### 2.4 Statistics

All variables were tested in 17 females in which differences in sound characteristics between burst types could be recognized unequivocally in all croaking sounds. In females 18 and 19 we could not recognize and analyze two different burst types unequivocally. Variables were tested for normal distribution using the Shapiro–Wilk test. If data were normally distributed, an unpaired t‐test was calculated to compare characteristics between sound bursts produced by pectoral fins in each individual. If data were not normally distributed, a Mann–Whitney *U* test was calculated. The first 10 croaking sounds produced by each individual were analyzed. In total, 170 croaking sounds and 968 double‐pulsed burst were analyzed.

In addition, exact binomial tests were run to determine whether the number of females in which pectoral sound bursts differed in a particular sound property was significantly larger than the number of individuals which lack such differences between fins. In those females in which two different types of sound bursts could not be recognized unequivocally in all croaking sounds analyzed, bursts were categorized as not differing in any sound characteristic. Accordingly, 19 females were analyzed in binomial tests. Binomial tests were calculated for each sound characteristic separately and for temporal and amplitude characteristics. Temporal characteristics refer to differences between pectoral fins either in burst periods or pulse periods or both (Figure 1b,c). Similarly, differences in amplitude characteristics refer to differences in peak‐to‐peak amplitudes between bursts produced by fins A and B or in the ratios of amplitudes of pulses 1 and 2 or both variables. All tests were calculated using SPSS 26 (IBM SPSS Statistics).

### 2.5 Ethical considerations

Dyadic contests between croaking gouramis consist of two phases: a lateral display phase followed by a frontal display phase. Croaking gouramis produce visual and acoustic signals only during the lateral display phase, without any physical contact between opponents (Ladich 1998). As the intention was to analyze signaling during contests, the agonistic interactions were stopped when contests proceeded to the frontal display phase during which fish bite each other. All applicable national and institutional guidelines for the care and use of animals were followed (permit numbers BMWFW‐66.006/0035‐WF/V/3b/2017; Animal Ethics and Experimental Board, Faculty of Life Science 2017‐010).

## 3 RESULTS

In 17 out of 19 females, differences between fin sites and therefore burst types were recorded in oscillograms and subsequently analyzed. Thus, the number of females in which characteristics of sound bursts produced by pectoral fins differed from each other was significantly higher (Binomial test: two‐tailed, *n* = 19, *p* < 0.01). Overall, the number of sound characteristics differing significantly between sound bursts varied from one (see F9, Tables 1 and 2) to all four characteristics (F8, Tables 1 and 2). Differences between fins in pulse periods or peak‐to‐peak amplitudes could be very pronounced (see oscillogram in Figure 1a) or less obvious (oscillogram in Figure 2).

### 3.1 Temporal characteristics of bursts

**Figure 3:**
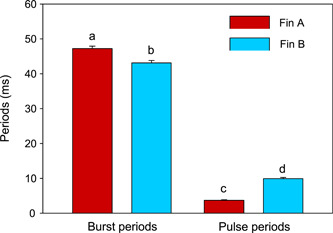
Exemplary analysis of temporal characteristics of double‐pulsed bursts in *Trichopsis vittata*. Mean (±SE) burst periods and pulse periods of bursts produced by pectoral fins A and B of one female (F8). An oscillogram of a croaking sound of this individual is shown in Figure 1a. Different lower case letters above bars indicate significant differences in sound characteristics between fins.

Burst periods differed significantly 14 out of 19 females (Table 1). The number of females in which burst periods differed from each other did not differ from the number of females where such differences were lacking (Binomial test: two‐tailed, *n* = 19, *p* = 0.064). Pulse periods differed in 15 out of 19 females. The number of females in which these periods differed between fins was significantly higher than the number of females in which this was not the case (Binomial test: two‐tailed, *n* = 19, *p* = 0.019). Figure 3 provides an example of significant differences in burst periods and pulse periods in an individual. In summary, 16 out of 19 individuals showed differences in temporal properties (either burst periods or pulse periods or both) of sound bursts produced by the different pectoral sonic organs, namely fin A and fin B (Binomial test: two‐tailed, *n* = 19, *p* = 0.004).

### 3.2 Amplitude characteristics of bursts

Relative peak‐to‐peak amplitudes of the first pulse within a burst and the ratio of peak‐to‐peak amplitude of the first and second pulse within a burst constitute the amplitude characteristics of bursts (see Figure 1c). Relative peak‐to‐peak amplitudes differed significantly between both fins in 7 out of 19 females (Table 2). This proportion of females was not significantly larger than the proportion lacking such differences (Binomial test: two‐tailed, *n* = 19, *p* = 0.064). Ratios of peak‐to‐peak amplitude of the first and second pulse within a burst differed in 8 out of 19 females. This proportion was again not significant (Binomial test: two‐tailed, *n* = 19, *p* = 0.648). In summary, amplitude characteristics of sounds do not differ between pectoral fin in the majority of females (12 out of 19) (Binomial test: two‐tailed, *n* = 19, *p* = 0.359).

Sound bursts produced by the left and right sonic organs in croaking gouramis differed in most females. Due to the small differences in sonic organs between sexes (Kratochvil, 1985; Ladich, 2015) and in sounds produced by both sexes (Ladich, 2007), we assume that similar differences could be found in males. The number of sound characteristics that differed between bursts generated by left and right pectoral fins varied from one (F9) to all four characteristics (F8). Note, however, that the production of different sound bursts by the left and right sonic organ (pectoral fins) in croaking gouramis may not represent a clear case of lateralized sound production in animals. Lateralized sound production in the strict sense means that one side of an animal or sonic organ produces a different sound type or sound serving another purpose than the other. As we were unable to clearly discriminate between the left and the right fin side, a definitive answer remains unsettled. There is a growing body of evidence that dolphins sound production is unilateral. The right pair of phonic lips generates echolocation clicks (bottlenose dolphin *Tursiops truncates*, false killer whale *Pseudorca crassidens*, beluga whale *Delphinapterus leucas*), whereas tonal sounds and other communication signals originate from the left pair (Ames et al., 2020; Madsen et al., 2013). In most birds, the tracheobronchial syrinx is a bipartite vocal organ in which each bronchus contains an independent sound source at its cranial end. Songbirds can sing with either side of the syrinx alone or with both sides simultaneously (Suthers, 1990, 2010). High frequencies are produced on the right side and low frequencies are generated on the left side; midrange frequencies can originate on either side. Independent production of sound bursts by the left and right pectoral fin is not possible in croaking gouramis because to produce sounds *T. vittata* has to beat its pectoral fins alternately. This is in contrast to the channel catfish, songbirds and toothed whales. The current results, therefore, point to a potential difference in the anatomical structures between fin sides and may constitute a new form of lateralized sound production (presupposed that the same fin side is always affected in the same manner).

**Table 1:** Mean temporal characteristics of bursts produced by fin A and fin B in female *Trichopsis vittata* (F1–F17)

	BPA (ms)	BPB (ms)	*p*	PPA (ms)	PPB (ms)	*p*
F1	43.35	43.90	8.808	7.56	5.15	**0.001**
F2	40.33	44.23	**0.001**	11.13	5.35	**0.001**
F3	49.20	45.75	0.102	14.17	14.08	0.568
F4	39.63	46.14	**0.001**	18.49	5.53	**0.001**
F5	46.98	41.03	**0.001**	6.59	6.39	0.163
F6	46.15	49.82	**0.001**	6.02	8.37	**0.001**
F7	43.9	43.4	**0.001**	9.33	6.14	**0.001**
F8	43.12	47.21	**0.001**	9.93	3.72	**0.001**
F9	44.77	44.88	0.934	8.87	9.77	**0.001**
F10	40.51	45.16	**0.036**	9.99	8.96	**0.001**
F11	44.41	48.49	**0.002**	8.30	7.88	**0.001**
F12	41.88	47.69	**0.001**	10.20	7.57	**0.001**
F13	49.23	45.72	**0.025**	7.01	9.69	**0.001**
F14	38.38	44.19	**0.001**	9.13	6.52	**0.001**
F15	46.57	42.94	**0.002**	6.84	7.55	**0.001**
F16	46.57	41.72	**0.001**	12.90	10.89	**0.001**
F17	45.33	42.31	**0.042**	9.18	8.22	**0.001**

*Note*: The temporal characteristics constitute the burst period of fin A (BPA) and fin B (BPB) and the pulse period of fin A (PPA) and fin B (PPB). Bold *p*‐values indicate significant differences between bursts produced by pectoral fins A and B in each female.

### 3.3 Temporal characteristics of sounds

Temporal sound properties differed between fins in a large proportion of females. Differences in burst periods between fins probably reflect small differences in the sonic motor patterns generated in the left and right pectoral fin motoneurons in the hindbrain of *T. vittata* (Ladich & Fine, 1992). Motoneurons responsible for sound production are activated by higher vocal nuclei in various regions of the brain (Bass et al., 2015; Ladich & Bass, 2011). While this has mainly been studied in toadfishes, in particular in the midshipman (*Porichthys notatus*), similar vocal pathways probably exist in other fish taxa including labyrinth fishes. Note, however, that a major difference exists between croaking gouramis and toadfishes: sonic muscles in toadfishes are used only for sound production, while pectoral fins serve multiple functions in croaking gouramis (genus *Trichopsis*), namely swimming, hovering and sound production. This is analogous to multiple functions of pectoral fins in catfishes (Fine & Ladich, 2003).

**Table 2:** Mean relative amplitude characteristics of bursts produced by fin A and fin B in female *Trichopsis vittata* (F1–F17)

	PtPA	PtPB	*p*	PtPA1/2	PtPB1/2	*p*
F1	0.266	0.294	0.279	1.380	1.434	**0.009**
F2	0.259	0.211	0.135	1.872	1.531	**0.001**
F3	0.312	0.375	0.070	1.393	1.552	**0.009**
F4	0.236	0.236	0.420	3.791	1.709	**0.007**
F5	0.553	0.722	**0.014**	1.636	2.155	1.000
F6	0.813	0.676	0.099	1.689	1.707	0.099
F7	0.502	0.431	0.099	1.611	1.629	0.254
F8	0.832	0.537	**0.001**	1.534	1.436	**0.001**
F9	0.403	0.427	0.378	1.348	1.334	0.353
F10	0.661	0.621	**0.001**	1.338	1.254	0.805
F11	0.603	0.595	0.759	1.447	1.364	**0.013**
F12	0.476	0.424	**0.005**	1.603	1.243	0.268
F13	0.152	0.150	0.991	1.508	1.384	**0.001**
F14	0.214	0.171	**0.005**	1.617	1.562	0.268
F15	0.149	0.139	0.082	1.822	2.018	**0.001**
F16	0.062	0.068	0.420	1.958	2.082	0.281
F17	0.068	0.067	0.949	1.956	2.001	0.073

*Note*: The amplitude characteristics constitute the relative peak‐to‐peak amplitude of bursts of fin A (PtPA) and fin B (PtPB) and the ratio between the relative peak‐to‐peak amplitudes of the first pulse and second pulse within bursts of fins A and B (PtPA1/2 and PtPB1/2). Bold *p*‐values indicate significant differences between sound bursts produced by pectoral fins A and B.

**Figure 4:**
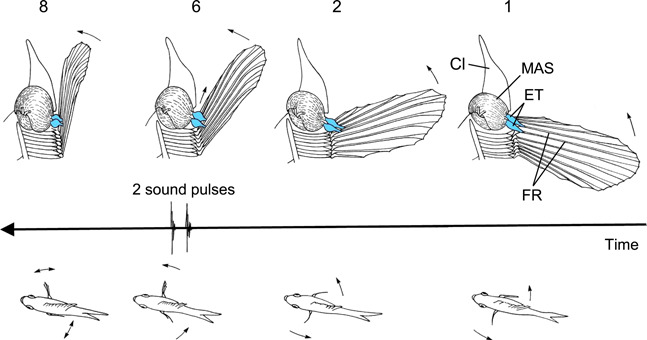
Pectoral tendon plucking mechanisms and sound generation in the croaking gourami. The upper row shows a medial view of the cleithrum (Cl), the enlarged superficial adductor muscle (MAS) and two enhanced tendons (ET) inserting on fin rays 5 and 6 (FR). The lower row represents 4 frames from a film (180 frames/s) showing the movement of the pectoral fins from a dorsal view. The oscillogram between two such rows illustrates the moment of sound emission during forward movement of the right fin. Due to muting of the left side, both pulses of a sound burst could only be generated by the right fin. Frames were numbered from right to left. Modified after Kratochvil (1985).

In contrast to burst periods, differences in pulse periods are hypothesized to reflect anatomical differences of the enhanced tendons in the left and right pectoral fin. Typically, two pulses are produced during forward movement of one pectoral fin because these fins possess two enhanced tendons which are stretched and pulled over a bony elevation of fin rays during sound production (Kratochvil, 1978). This almost simultaneous plucking of two tendons yields two pulses per fin and a series of two‐pulsed bursts (termed croaking sound) when both fins are beaten alternately (Figure 4). The distances between spread fin rays and subsequently enhanced tendons may vary slightly, resulting in fin‐typical pulse periods which almost always (15 out of 19 females) differ between fins.

### 3.4 Amplitude characteristics of sounds

The peak‐to‐peak amplitudes of pulses within sound bursts probably depends on the diameter of the enhanced tendons or perhaps on differences in stretching enhanced tendons by pectoral muscles (MAS in Figure 4). Kratochvil (1980) demonstrated that the amplitude of the first pulse is approximately twice as large as that of the second pulse in male pygmy gourami *Trichopsis pumila*. According to that author, this is due to the much larger diameter of the first versus second enhanced tendon. Recent measurements showed that the diameter of the first tendon is approximately 1.5 times that of the second in both sexes (females: 0.17 vs. 0.11 mm; males: 0.35 vs. 0.24 mm) of *T. pumila* (Liesch & Ladich, 2020). While the current study detected differences in peak‐to‐peak amplitudes of first pulses of bursts in 5 out of 19 females and differences in amplitude ratios between the first and second pulse within bursts in 8 out of 19 females, this represent less than half of the individuals. This is in contrast to temporal sound characteristics, in particular pulse periods, which differed significantly (16 out of 19 females).

### 3.5 Lateralization of sound production in fishes

Among fishes, lateralization of sound production has been reported only in the channel catfish. Fine et al. (1996) argued that channel catfish are in some way right‐handed because they produce more stridulation sounds with their right pectoral fin (by abducting the fin and rubbing a dorsal ridged process of the enhanced first fin ray (pectoral spine) in a groove of the shoulder girdle). Right‐handedness is dominant over left‐handedness (similar to humans) not just in the number of sounds but perhaps also in sound duration.

The question arises whether swimbladder drumming muscles of the left and right side may contract asynchronously and thus exhibit different pattern similar to gouramis. Fine (1982) described a possible lateralization based on three sounds recorded in the field, and evoked by electrical stimulation in the laboratory, in the oyster toadfish. Later work demonstrated that these unusual sounds were caused by a nearby fish calling over the calls of the first fish (acoustic tagging) (Thorson & Fine, 2002). Bass and Baker (1991) and Connaughton (2004) observed that asynchronous firing of sonic nerves results in asynchronous contraction of the sonic muscle in the northern sea robin (*Prionotus carolinus*, Fam. Triglidae). Except in the northern sea robin, in all other fish species investigated (piranhas *Pygocentrus* spp., the plain midshipman, the oyster toadfish *O. tau*, the longhorn sculpin *Myoxocephalus scorpius*, the Pacific staghorn sculpin *Leptocottus armatus*) sonic nerves fire synchronously (Bass & Baker, 1991; Kastberger, 1981). It remains to be clarified whether asynchronous firing of sonic nerves affects the characteristics of swim bladder sounds. Asynchronous firing will potentially increase the fundamental frequency of swim bladder sounds but it is unlikely that different sounds will be emitted on the left and right side of the body. Thus, in contrast to toothed whales and songbirds, no differences in sound types or function of sounds have been found in fishes so far.

## 4 CONCLUSION

Current data demonstrate that the characteristics of sounds produced by the left and right sonic organ in the fish species *T. vittata* differed significantly in most specimens. These differences in sound properties may constitute a form of lateralized sound production in vocal fishes. A recent survey showed that vocalizing behavior is known in 175 out of 470 families of ray‐finned fishes (Actinopterygi: 34,000 extant species). Among these fishes, pectoral fin‐based sonic mechanisms are known in at least 15, mostly siluroid (catfish) families (Rice et al., 2022). This makes it likely that forms of lateralized sound production are more widespread in fishes. It remains to be clarified whether differences between sound generated by the left and right side have a function, such as in tetrapods (toothed whales and songbirds).

## AUTHOR CONTRIBUTIONS

**Isabelle Pia Maiditsch**: experiments, experiment design, data analysis and statistics, draft. **Friedrich Ladich**: experiment design, data analysis, and statistics, interpretation of results, draft. All authors have seen and approved the manuscript, and the manuscript has not been accepted or published elsewhere.

## CONFLICTS OF INTEREST

The authors declare no conflicts of interest.

## Data Availability

The data that support the findings of this study are available from the corresponding author upon reasonable request.
